# Enhanced Control of the Fungus Gnat *Bradysia odoriphaga* (Diptera: Sciaridae) by Co-Application of Clothianidin and Hexaflumuron

**DOI:** 10.3390/insects12070571

**Published:** 2021-06-22

**Authors:** Yongqing Wang, Kai Wan, Ruifei Wang, Jiyingzi Wu, Ruiquan Hou, Kunyu Zhao, Zhixiang Zhang, Jianjun Chen, Dongmei Cheng

**Affiliations:** 1 College of Agriculture and Biology, Zhongkai University of Agriculture and Engineering, Guangzhou 510225, China; wyqzdsys@163.com; 2 Key Laboratory of Natural Pesticide and Chemical Biology, Ministry of Education, South China Agricultural University, Guangzhou 510642, China; wkaizx@aliyun.com (K.W.); ruifeiwangny@163.com (R.W.); wjyzzdsys@163.com (J.W.); gdlzhrq@163.com (R.H.); zky849822807@163.com (K.Z.); zdsys@scau.edu.cn (Z.Z.); 3 Institute of Quality Standard and Monitoring Technology for Agro-Products of Guangdong, Academy of Agricultural Sciences, Guangzhou 510642, China; 4 Department of Environmental Horticulture and Mid-Florida Research and Education Center, Institute of Food and Agricultural Sciences, University of Florida, Apopka, FL 32703, USA

**Keywords:** absorption and dissipation, accumulation factor, chive, clothianidin, fungus gnats, hexaflumuron

## Abstract

**Simple Summary:**

The fungus gnat (*Bradysia odoriphaga* Yang and Zhang) is a major pest of chive (*Allium tuberosum* Rottl. ex Spreng) that can cause more than 50% yield losses during chive production in China. The neonicotinoid, neuroactive insecticide clothianidin has been widely used for chive gnat control; however, following intensive use of this compound, its effects on chive gnat have been markedly reduced, possibly due to the development of insecticide resistance. Hexaflumuron is an insect growth regulator which disrupts chitin synthesis during molting by inhibiting the incorporation of N-acetyl glucosamine monomers into the integument chitin of insects. The present study shows that co-drenching of clothianidin and hexaflumuron enhanced chive absorption of clothianidin, resulting in significant improvement in control of fungus gnat. Additionally, the terminal residues of clothianidin in chive were lower than the maximum residue limit in chive set by the Codex Alimentarius Commission, hence, the chive could be safe for consumption.

**Abstract:**

The fungus gnat is a major pest of chive in China. Its control has been relied heavily on the application of clothianidin. Due to the intensive application, its control efficacy become reduced. The present study was intended to evaluate co-drenching of clothianidin with hexaflumuron on absorption and dissipation of clothianidin in chive plants and soils and determine the effect of such application on control efficacies. Chive production fields in Guangdong and Hubei Provinces were drenched with clothianidin alone and a mixture of clothianidin and hexaflumuron at low application rates. Concentrations of clothianidin in chive plants and soils were analyzed by HPLC. Results showed that co-application had higher control efficacies against the fungus gnat than clothianidin alone. The co-application enhanced clothianidin absorption and dissipation and extended the half-lives of clothianidin in chive. It was likely that hexaflumuron protected chive roots from larva damage, and healthy roots absorbed more clothianidin, resulting in the extension of the half-lives. Additionally, the terminal residues of clothianidin in chive after 14 days of application were lower than the maximum residue limit in chive set by the Codex Alimentarius Commission. This study for the first time documented that co-application of clothianidin and hexaflumuron improved chive plants in absorption and dissipation of clothianidin and enhanced fungus gnat control efficacies.

## 1. Introduction

Chive (*Allium tuberosum* Rottl. ex Spreng) is a perennial herbaceous vegetable that has high nutritional and economic value in Eastern Asia [1]. It is native to China, commonly known as Chinese chive and has a long history of cultivation. The plant grows by expanding perennial rhizomes but also readily sprouts from seeds. Its green leaves are flat and grass-like about 35 cm long and 0.8 cm wide. Shoots are cut during the growing season as fresh-leaved vegetable and has garlic like flavor. New shoots will emerge and can be cut again several times. In addition to being used as fresh vegetable, Chinese chive has also been used as a medicinal plant [2].

The fungus gnat, *Bradysia odoriphaga* Yang and Zhang (Diptera: Sciaridae), is the most destructive pest of *Allium* crops, particularly Chinese chive. Thus, it commonly known as chive gnat [3]. Female gnats lay eggs around the roots of chive in soil, and larvae directly damage roots, impeding root absorption of water and nutrients and thus plant growth [4]. The chive gnat produces overlapping generations annually in the chive fields with peak damage occurring in spring and autumn. It enters dormancy in winter. *B. odoriphaga* attacks 20–30% of chive fields and causes almost 50% yield losses in China [5,6].

Chive gnat larvae are typically controlled using insecticides, such as organophosphates, carbamates, and neonicotinoids applied as root drench although biological control using entomopathogenic nematodes has been shown to be an alternative method [7]. Clothianidin (Celero; Arvesta Corp., San Francisco, CA, USA), is a second-generation neonicotinoid possessing stomach and contact activity, and operates by interfering with the nicotinic acetylcholine receptors in the insect nervous system [8]. Clothianidin has been widely used for control of *B. odoriphaga* [9]. However, due to the intensive use of this insecticide, its control efficacy has been markedly reduced. A study on several field populations of *B. odoriphaga* in China showed that they developed low to moderate levels of resistance to clothianidin [10]. As a result, much higher application rates, ranging from 3 to 6 kg ai/ha have been used [9]. The increased application of clothianidin may pose considerable risk to the environment, human health, and beneficial fauna [11].

To pursue sustainable pest management, there is an urgent need for reducing application rates of clothianidin and also improving its control efficacy. A recent study showed that application of clothianidin in combination with other insecticides improves control efficiency against *Anopheles gambiae* populations [12]. In addition to insecticides, insect growth regulators have been used for controlling fungus gnats [13]. Hexaflumuron is an insect growth regulator [14] that disrupts chitin synthesis during molting by inhibiting the incorporation of N-acetylglucosamine monomers into the integument chitin of insects including subterranean termite species [15]. Hexaflumuron has been shown to reduce the growth rate, adult emergence rate, and fecundity of the *B. odoriphaga* [16]. A mix of clothianidin and hexaflumuron has been used for controlling *B. odoriphaga* [17]. However, there has been no information on the effect of the co-application of hexaflumuron and clothianidin on the dissipation and absorption of clothianidin in chive and soil.

The objectives of this study were to evaluate the dynamics of clothianidin in chive plants and soils, analyze its terminal residue in soil and chive, and determine if co-application of clothianidin and hexaflumuron could improve chive gnat control efficacies.

## 2. Materials and Methods

### 2.1. Chemicals and Reagents

Clothianidin standard (99.5%) was purchased from Dr. Ehrenstorfer (Augsburg, Germany). A 30% suspension concentrate (30% SC) containing 25% of clothianidin and 5% of hexaflumuron was manufactured by Boshiwei Biotechnology Co., Ltd., (Haikou, China). A 48% suspension concentrate (48% SC) containing 48% of clothianidin was manufactured by Zhaoyuan Sanlian Chemical Co., Ltd., (Yantai, China). Analytical grade acetonitrile n-hexane, acetone, and sodium chloride were purchased from Tianjin Fuchen Chemical Reagent Factory Co., Ltd. (Tianjin, China). Solid phase extraction (SPE) column (florisil, 1 g/6 mL) and HPLC grade methanol were purchased from ANPEL Laboratory Technologies Co., Ltd. (Shanghai, China).

### 2.2. Plant Material

Seeds of chive cultivar Fujiu No. 8 were purchased from Fusheng Co., Ltd. (Zhengzhou, China). Seeds were sown in April 2018. Seedlings when growing up to 10 cm were transplanted to multiple field plots (20 m^2^) located in Guangdong Province, Southern China and Hubei Province, Central China in June 2018. There were 800 seedlings per plot, and each plot was separated by a 2 m buffer area. Plant production was managed according to recommended agronomic practices [18]. Three field experiments were conducted in the mentioned two locations, respectively.

### 2.3. Field Experiments for Studying Dynamics of Clothianidin, Its Residue in Soil and Chive, and Control Efficacies

The first experiment was to study the dynamics of clothianidin, i.e., its absorption and dissipation in chive and soils. Six field plots at each location were randomly selected. After one month of transplanting, plants grown in three randomly selected plots at each location were subjected to drench of roots with 30% SC at a rate of 1350 g.a.i.ha^−1^, and those grown in the remaining three plots were drenched with 48% SC at a rate of 1350 g.a.i.ha^−1^ as the control treatment. Each treatment had three replicated plots. Subsequently, 1 kg chive (shoots, i.e., above ground leaves with triangular bases) and soil samples were randomly collected from each of the treatment plots at 2 h (0 d), 1, 2, 3, 5, 14, and 21 d after the chemical application. The soil was sampled from randomly selected 6 to 12 points per plot in a depth ranging from 0 to 10 cm. All samples were stored at −20 °C before sample preparation and HPLC analysis. To determine the absorption of clothianidin in chive shoots, accumulation factor (AF) was used to express clothianidin absorbed by chive in relation to clothianidin in soils:

AF = C_chive_/C_soil_

where $C_{\mathrm{chive}}$ and $C_{\mathrm{soil}}\;$ were concentrations of clothianidin in chive shoots and soil, respectively.

The second experiment was conducted for analyzing the terminal residue of clothianidin in soil and chive. Twenty-four field plots were selected per location. Roots of plants grown in randomly selected six plots were drenched with 30% SC and 48% SC, three plots each, at 675 g.a.i.ha^−1^ twice, and those grown in another randomly selected six plots were drenched at 675 g.a.i.ha^−1^ three times with a 7-d interval. Similar drench treatments were performed on roots of plants grown in the remaining 12 plots with 30% SC and 48% SC, each at 1350 g.a.i.ha^−1^ twice and three times, three plots each, at a 7-d interval, respectively. The application dosage was computed based on amount of clothianidin. More than 1 kg of chive and soil samples were randomly collected per plot after 7, 14, and 30 days of drenching. All samples were stored at −20 °C before sample preparation and HPLC analysis.

The third field experiment was performed to determine effects of 30% SC or 48% SC on control of *B. odoriphaga*. A total of 15 field plots were selected at each location. Six randomly selected plots at each location were drenched with 30% SC at 675 and 1350 g.a.i.ha^−1^, three plots each, another six plots were drenched with 48% SC at the same rates as 30% SC, also three plots each, respectively. The remaining three plots at each location were drenched with water as the control. All field experiments in the two locations were arranged as a completely randomized design with three replications.

### 2.4. Evluation of Control Efficacy

To evaluate the control efficacy in the third field experiment, the numbers of *B. odoriphaga* around the pseudostems/bulbs and roots on randomly selected chive plants (nine locations and a Z-shaped sampling for every repetition, 20 cm × 20 cm for each quadrat) were counted at 0, 1, 3, 5, 7, 14 and 30 days after the applications. The observed mortality and corrected mortality for each treatment were calculated by following Equations (1) and (2), respectively:

(1) $$\mathrm{Observed}\;\mathrm{mortality}\;\left(\%\right)=\frac{\mathrm{Initial}\;\mathrm{number}\;\mathrm{of}\;\mathrm{live}\;B.\;odoriphaga-\;\mathrm{number}\;\mathrm{of}\;\mathrm{live}\;B.\;odoriphaga\;\mathrm{after}\;\mathrm{treatment}}{\mathrm{Initial}\;\mathrm{number}\;\mathrm{of}\;\mathrm{live}\;B.\;odoriphaga}\times 100$$

(2) $$\mathrm{Corrected}\;\mathrm{mortality}\;\left(\%\right)=\frac{X_{i}\;-\;X_{\mathrm{control}}}{100-\;X_{\mathrm{control}}}\times 100$$

where X_i_ was the observed mortality from Equation (1) after a specific treatment in a location, and X_control_ was the mortality from Equation (1) after water was applied in the same location.

### 2.5. Sample Preparation

Collected chive samples, 30 g each, were homogenized and placed into 100 mL centrifuge tubes. Then, 10 mL of 10% phosphoric acid and 50 mL of acetonitrile were added as described by Lv et al. [19]. The mixture was subjected to ultrasonic treatment for 30 min and centrifuged at 4000 r/min for 5 min. The extracts were filtered with a filter paper into 100 mL graduated cylinders containing 10 g sodium chloride. The graduated cylinder was covered and shaken for 1 min and allowed to rest for 30 min. About 25 mL supernatant was transferred into a 150 mL flat bottom flask and evaporated to almost dryness.

The concentrated sample was purified with a Florisil column [20] as follows: First, Florisil was successively activated with 5 mL *n*-hexane/acetone (*v/v* = 90/10) and 5 mL of *n*-hexane. Second, the evaporated samples were dissolved twice in 10 mL *n*-hexane/acetone (*v/v* = 90/10) and transferred completely to the column. Impurities were then eluted by 10 mL *n*-hexane/acetone (*v/v* = 85/15), and the sample was eluted by 20 mL *n*-hexane/acetone (*v/v* = 70/30). The collected eluent (50 mL) was dried at 40 °C and then diluted with chromatographic grade methanol to 3 mL. This solution was filtered through a 0.22 μM Teflon membrane filter and analyzed by HPLC.

Air dried soil (10 g) was shaken with acetonitrile (50 mL) in a 100 mL centrifuge. The suspensions were ultrasonically extracted for 30 min and then centrifuged at 4000 r/min for 5 min. A 25 mL aliquot of the supernatant was transferred to a 50 mL flat-bottomed flask and evaporated until nearly dry using a rotary evaporator. Finally, this solution was filtered through a 0.22 μM Teflon membrane filter and analyzed by HPLC.

### 2.6. HPLC Analysis

A LC-20A HPLC system (Shimadzu, Kyoto, Japan) equipped with a variable wavelength UV-visible detector was used for clothianidin analysis. Chromatography was performed using a Zorbax SB-C_18_ column (250 mm × 4.6 μM, 5 μM, Agilent, Santa Clara, CA, USA) [21]. The mobile phase was methanol/water (*v/v* = 30/70), and the flow rate was 1 mL/min. The injection volume was 10 μL, and the residues were determined at 265 nm. The retention time of clothianidin was approximately 12.5 min.

Standard solutions of clothianidin were dissolved with chromatographic grade methanol at the five concentrations: 0.01, 0.05, 0.1, 0.5, 1, and 5 mg/L. Linear calibration graphs were constructed by least-squares regression of concentration versus peak area of calibration standards.

For recovery assay, the untreated chive and soil samples were fortified with appropriate amounts of standard stock solutions to reach specific concentrations (0.1, 1.5, and 2.0 mg/L). The recovery rates (relative) were calculated via the matrix-matched calibration curves to determine the amount of clothianidin residue in chive leaves and soil, respectively. All the recovery experiments were conducted in five replicates.

### 2.7. Data Analysis

The dissipation kinetics of the residue data was evaluated by fitting the dynamic degradation models to the data. The residue results of all samples were analyzed using nonlinear regression. Data were presented as mean ± standard error (S.E.) with three replications. All data were subjected to analysis of variance using Microsoft Office Excel 2010 and the SPSS software platform v. 25.0 (IBM Corporation, Somers, NY, USA). If significance occurred among means, which were separated by Tukey’s honestly significant difference (HSD) test at *p* < 0.05 level.

## 3. Results

### 3.1. Method Validation

Using an external standard solution of clothianidin, linear calibration graphs were constructed by least-squares regression of concentration versus peak area of calibration standards. Good linearity of matrix-matched calibration was obtained, with 0.01~5.00 mg/L (Y = 43784X − 1359.1 with R^2^ = 0.9999). The mean recoveries and relative standard deviations for the untreated chive were 87.08% and 3.92% and for untreated soil samples (red soil) were 93.45% and 7.11%. The limit of detection (LOD) of the method was 0.01 mg/L at an S/N ratio of 3 in chive and soil.

### 3.2. Absorption of Clothianidin in Chive

**Figure 1:**
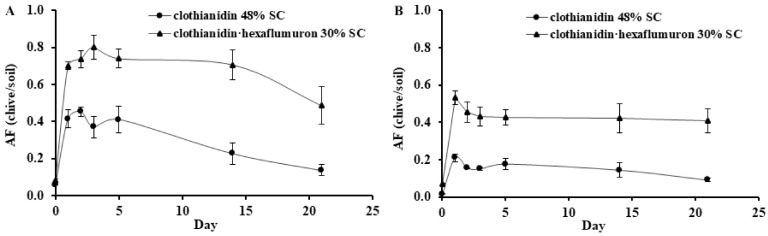
Accumulation factors (AFs) of clothianidin in shoots of chive plants grown in Guangdong (**A**) and Hubei (**B**) soils after roots were drenched with 48% SC (clothianidin only) and 30% SC (clothianidin plus hexaflumuron) at a rate of 1350 g.a.i.ha^−1^. Each point represents the mean ± SE (*n* = 3). AFs at each sampling day were significantly different between the two treatments based on Tukey’s HSD test at *p* < 0.05 with the exception of day 0.

Data for chive absorption of clothianidin were analyzed based on AF, which is shown in Figure 1 by plotting the AF values against time. Results showed that clothianidin in chive shoots significantly increased after applications. As shown in Figure 1A,B the AF values across the experimental duration were much higher in chive shoots applied with 30% SC than those applied with 48% SC regardless of production location.

### 3.3. Dissipation of Clothianidin in Chive

**Figure 2:**
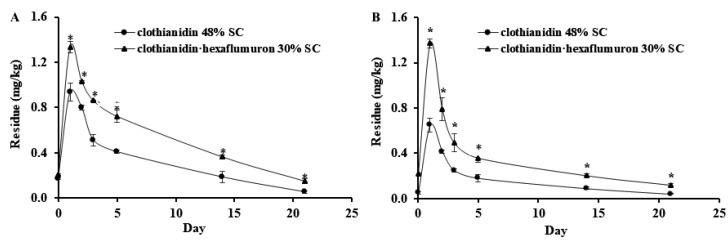
Absorption and dissipation of clothianidin in shoots of chive plants grown in Guangdong (**A**) and Hubei (**B**) by root drenched with 48% SC (clothianidin only) and 30% SC (clothianidin plus hexaflumuron) at a rate of 1350 g.a.i.ha^−1^. Each point represents the mean ± SE (*n* = 3) where * indicates significant differences between the two treatments based on Tukey’s HSD test at *p* < 0.05 with the exception of day 0.

The dynamics of clothianidin in chive shoots in Guangdong and Hubei are presented in Figure 2. Clothianidin in chive shoots increased at 2 h after application and then decreased over time regardless treatments and experimental locations.

The initial values of clothianidin applied as 30% SC in chive plants grown in Guangdong were 0.209 mg/kg (Figure 2A) and 0.222 mg/kg in Hubei (Figure 2B). Clothianidin concentrations in chive shoots of plants grown in Guangdong and Hubei reached the highest levels at 1.334 and 1.373 mg/kg on day one after application and then decreased to 0.148 and 0.119 mg/kg with dissipation rates of 88.9% and 91.3%, respectively.

The initial values of clothianidin applied as 48% SC in shoots of chive plants grown in Guangdong and Hubei were 0.173 (Figure 2A) and 0.051 mg/kg (Figure 2B), respectively. The maximum values of clothianidin occurred on day 1 at 0.936 mg/kg in chive plants grown in Guangdong and 0.649 mg/kg in those grown in Hubei. Subsequently, clothianidin concentrations decreased to 0.052 and 0.041 mg/kg in chive grown in the two locations with dissipation rates of 94.4% and 93.7%, respectively.

Clothianidin concentrations in chive shoots treated with 30% SC over the experimental period were significantly higher than those treated with 48% SC, regardless of growing location.

### 3.4. Dissipation of Clothianidin in Soil

**Figure 3:**
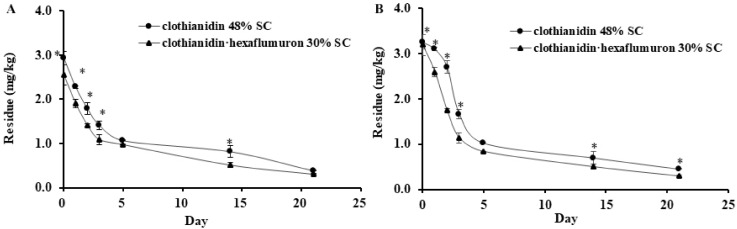
Adsorption and dissipation of clothianidin in soil in Guangdong (**A**) and Hubei (**B**) by root drenched with 48% SC (clothianidin only) and 30% SC (clothianidin plus hexaflumuron) at a rate of 1350 g.a.i.ha^−1^. Each point represents the mean values of three experiments ± SE (*n* = 3), and * indicates significant differences between the two treatments at the same time point based on Tukey’s HSD test at *p* < 0.05.

The dynamics of clothianidin in chive-grown soils in Guangdong and Hubei are presented in Figure 3. Irrespective of chemicals and locations, the residues of clothianidin in soil were the highest right after application and then rapidly decreased from 2 h to day 5, the decrease became much slow thereafter. On day 21, the residue of clothianidin applied with 30% SC in Guangdong and Hubei soil samples were 0.315 and 0.294 mg/kg, respectively, with the respective dissipation rates of 87.6% and 90.8%. Similarly, soil residue levels of clothianidin applied as 48% SC were 0.398 mg/kg in Guangdong soil and 0.449 mg/kg in Hubei soil with respective dissipation rates of 86.7% and 86.2%. Clothianidin residue in soil treated with 48% SC was generally higher than soil treated with 30% SC regardless of location.

The dissipation equations and half-lives of clothianidin in chive and soil are shown in Table 1. The half-lives of clothianidin in chive samples from Guangdong and Hubei after application of 30% SC were 6.93 and 6.73 days, respectively, and the half-lives were 7.87 and 6.73 days in soil of the two locations. The half-lives of clothianidin in chive samples from Guangdong and Hubei after application of 48% SC were 5.21 and 5.73 days respectively, and 8.45 and 7.45 days in soil, respectively.

### 3.5. Terminal Residue of Clothianidin in Chive and Soil

The terminal residues of clothianidin in shoots of chive plants produced in Guangdong and Hubei are presented in Table 2. Chive plants drenched with 30% SC had higher clothianidin residue on day 7 irrespective of application rates and times, ranging from 0.205 to 2.227 mg/kg in Guangdong and 0.191 to 0.568 mg/kg in Hubei. The levels of residue then decreased thereafter. On day 30, residues varied from <LOD to 0.459 mg/kg in Guangdong and <LOD in Hubei. Clothianidin residue levels in soil were initially higher, and then decreased to levels above LOD. In general, soil clothianidin levels were lower than chive grown in Guangdong, whereas clothianidin residue levels in soil of Hubei were higher than those in chive grown there.

The terminal residues of clothianidin in chive plants drenched with 48% SC had similar trends as those applied with 30% SC. The concentrations of clothianidin residue in chive and soil were largely comparable to those treated with 30% SC.

### 3.6. Control Efficiencies

The corrected mortalities or control efficiencies of *B. odoriphaga* resulting from the application of 30% SC or 48% SC, each with two rates, are presented in Table 3.

Generally, with increased application rates, the corrected mortalities increased. After application, the control efficacies gradually increased from day 1 to day 30. Thus, the highest control efficacies occurred on day 30. By comparison, application of 30% SC showed higher control efficacies against *B. odoriphaga* than 48% SC regardless of the location. More importantly, the control efficiencies were not significantly different between the two application rates of 30% SC starting from day 3 in Guangdong and day 5 in Hubei locations. However, significant differences occurred between the two rates of 48% SC on most of sampling days in Guangdong but not in Hubei.

## 4. Discussion

Chinese chive is an important fresh vegetable in Asian counties. Fungus gnat has been a notorious problem in chive production. After identifying the effectiveness of clothianidin in control of chive gnat [22], this insecticide has been extensively used. The heavy use of the same insecticide in chive has resulted in the development of resistance to clothianidin in *B. odoriphaga* and a great reduction of control efficacies in China [10]. The reduced efficacies subsequently lead to increased application rates [9], which may pose potential risks to non-target organisms and contamination of the environment [11]. To improve control efficacies, a recent study showed that clothianidin in combination with hexaflumuron could improve gnat control [17]. However, the dynamics of clothianidin in co-application with hexaflumuron have not been investigated.

The present study evaluated the absorption and dissipation of clothianidin in chive produced in two different regions, its residue in chive and soils, and the control efficacies for *B. odoriphaga* after it was applied alone or with hexaflumuron. Our results showed that the half-lives of clothianidin in chive were longer (Table 1) and clothianidin concentrations in chive shoots (Figure 1 and Figure 2) were higher when it was applied in combination with hexaflumuron than when applied alone, regardless of the location. The increased absorption and half-lives could explain the increased control efficacies. The synergistic effects resulting from the combined application are probably due to the following reasons: Hexaflumuron inhibited the hatching rate of the gnat’s eggs and decreased the survival rate of newly hatched larvae, protecting chive roots from gnat damage. Healthy roots should be able to take up more clothianidin, resulting in the higher clothianidin in plants and increased control efficiencies. Healthy root systems could readily absorb water and nutrients from soils for enhanced plant growth. Additionally, the combination of insecticides may affect physiological status of plants, causing a slow breakdown of clothianidin in plants.

This study also documents that the terminal residue of clothianidin in chive shoots were below the maximum residue limit (MRL) set by the European Union (1.50 mg/kg) 14 days after application at the rates of 675 or 1350 g.a.i.ha^−1^. Commercially, chive shoots are harvested every 20 to 30 days depending on growth temperature and soil fertility [23,24]. Thus, chive leaves treated with clothianidin alone or with hexaflumuron at the aforementioned rates are safe for consumption.

The control efficiency of *B. odoriphaga* after the application of the mixture of clothianidin and hexaflumuron was better than the use of clothianidin alone (Table 3). The lower rate (675 g.a.i.ha^−1^) had similar control efficiencies as the higher rate (1350 g.a.i.ha^−1^), and the control duration on *B. odoriphaga* was also extended when the mixture was applied. Therefore, the application of the mixture could reduce the application rates and times. Agriculture has always been a central concern of human health and beneficial organisms, particularly pesticide application. The use the mixture of the two insecticides in chive production should improve chive gnat control efficacies by reducing application rates and/or frequency, which may benefit to the environment and natural enemies and improve chive production.

It is worth to note that the dissipation of clothianidin in the soil of Guangdong was different from that of Hubei, i.e., clothianidin residues in Guangdong soil were lower compared to chive; the opposite was true for chive grown in Hubei soil (Table 2). This is probably related to soil properties and weather conditions, primarily temperature. The soil in Guangdong is red soil with a pH about 5.7, the average content of soil organic matter in Guangdong was 11.2 g/kg. Average temperatures in Guangdong range from 7 °C in the winter to 34 °C in the summer. The soil in Hubei is yellow-brown soil with a pH about 6.8, the average content of soil organic matter in Hubei was 6.73 g/kg. Average temperatures vary from −25 °C in the winter to 27 °C in the summer [25,26]. The higher temperature in Guangdong promoted chive growth and concomitantly increased uptake of clothianidin by chive. On the other hand, relatively lower temperatures in Hubei might result in comparably slower growth and reduced uptake clothianidin. Additionally, soil pH and organic content could also affect clothianidin bioavailability in soil [24]. Nevertheless, our study suggests that clothianidin application rates may need to be adjusted based on plant growth environments and soil conditions.

## 5. Conclusions

This study evaluated the absorption and dissipation of clothianidin in Chinese chive applied through drenching of clothianidin only or in combination with hexaflumuron into roots of chive plants produced in Guangdong and Hubei provinces, China. The terminal residue in chive shoots and soils was analyzed, and chive gnat control efficacies were evaluated. The results showed that the co-application increased control efficacies and clothianidin absorption and dissipation in chive compared to clothianidin application alone. The half-lives of clothianidin were longer in chive and shorter in soil when applied in combination with hexaflumuron compared to clothianidin application only. The terminal residues of clothianidin in chive were below the MRL set by the European Union. Thus, clothianidin applied at rates of 675 or 1350 g.a.i.ha^−1^ was safe for consumption.

## Figures and Tables

**Table 1:** Dissipation equation and half-lives of clothianidin in chive shoots and soils.

Sample	Insecticide ^z^	Sample Location	Dissipation Equations	Correlation Coefficient (*R*^2^)	Half-Lives (Day)
Chive	Clo + Hex	Guangdong	C = 1.2923 × 10^−0.100T^	0.9829	6.93
	Clo + Hex	Hubei	C = 0.8949 × 10^−0.103T^	0.8593	6.73
	Clo	Guangdong	C = 0.9371 × 10^−0.133T^	0.9718	5.21
	Clo	Hubei	C = 0.4748 × 10^−0.121T^	0.9181	5.73
Soil	Clo + Hex	Guangdong	C = 1.8386 × 10^−0.089T^	0.8758	7.87
	Clo + Hex	Hubei	C = 2.1715 × 10^−0.103T^	0.9753	6.73
	Clo	Guangdong	C = 2.2034 × 10^−0.082T^	0.9065	8.45
	Clo	Hubei	C = 2.6938 × 10^−0.093T^	0.8776	7.45

^z^ Roots were drenched with 30% SC (clothianidin plus hexaflumuron, i.e., Clo + Hex) or 48% SC (clothianidin only, i.e., Clo), each at a rate of 1350 g.a.i.ha^−1^ based on clothianidin.

**Table 2:** Terminal residues of clothianidin in chive shoots and soils of Guangdong and Hubei after drenching with a clothianidin and clothianidin mixture or clothianidin only at rates of 675 or 1350 g.a.i.ha^−1^ two and three times, respectively.

Sample	Dosage (g.a.i.ha^−1^)	Application Times	Time (d)	Residues (mg/kg)			
				Guangdong		Hubei	
				Clo + Hex	Clo	Clo + Hex	Clo
Chive	675	2	7	0.205 a	0.150 c	0.191 a,b	0.173 b
			14	0.185 a	0.107 b	<LOD	0.024 c
			30	<LOD	<LOD	<LOD	<LOD
	675	3	7	1.764 a	1.647 a	0.288 b	0.213 b
			14	0.236 a	0.254 a	0.020 b	0.019 b
			30	0.211 a	0.136 b	<LOD	<LOD
	1350	2	7	0.568 a	0.205 c	0.279 b	0.117 d
			14	0.303 a	0.085 c	0.172 b	0.023 d
			30	0.066 a	<LOD	<LOD	<LOD
	1350	3	7	2.227 a	1.864 b	0.568 c	0.460 c
			14	0.719 a	0.569 b	0.200 c	0.021 d
			30	0.459 a	0.259 b	<LOD	<LOD
Soil	675	2	7	0.192 c	0.214 c	0.510 b	0.624 a
			14	0.096 d	0.130 c	0.219 b	0.327 a
			30	0.023 b	0.027 b	0.080 a	0.074 a
	675	3	7	0.318 b	0.324 b	0.693 a	0.718 a
			14	0.041 c	0.062 b	0.682 a	0.678 a
			30	0.027 c	0.043 c	0.481 b	0.539 a
	1350	2	7	1.254 c	1.437 b	1.540 b	1.863 a
			14	0.120 c	0.716 a	0.490 b	0.514 b
			30	0.044 b	0.035 b	0.327 a	0.331 a
	1350	3	7	2.103 b	2.534 a	1.605 d	1.792 c
			14	0.185 c	0.140 d	1.526 a	1.439 a
			30	0.044 d	0.054 c	0.304 b	0.432 a

Note: Roots were drenched with 30% SC (clothianidin plus hexaflumuron, i.e., Clo + Hex) or 48% SC (clothianidin only, i.e., Clo). Different letters in each line indicates statistical differences based on Tukey’s HSD test at *p* < 0.05 level.

**Table 3:** The corrected mortalities (%) of *B. odoriphaga* resulting from application of clothianidin alone or with clothianidin at two rates in chive production field of Guangdong and Hubei Provinces.

Sites	Insecticide ^z^	Dosage (g.a.i.ha^−1^)	Corrected Mortality (%)					
			1 d	3 d	5 d	7 d	14 d	30 d
Guangdong	Clo + Hex	675	48.14 ± 1.32 b	57.85 ± 1.25 abc	70.83 ± 5.61 ab	85.61 ± 3.30 ab	90.84 ± 4.79 ab	94.03 ± 2.98 ab
		1350	58.50 ± 3.20 a	63.58 ± 1.94 a	75.37 ± 2.07 a	88.60 ± 3.25 a	94.64 ± 2.21 a	96.82 ± 1.58 a
	Clo	675	34.97 ± 2.12 cd	45.95 ± 2.60 de	49.39 ± 2.08 d	58.13 ± 1.48 d	64.18 ± 2.22 de	69.60 ± 2.13 d
		1350	40.68 ± 4.36 bc	58.91 ± 1.97 ab	62.94 ± 2.61 bc	68.62 ± 2.57 c	73.29 ± 2.40 cd	77.76 ± 1.87 c
Hubei	Clo + Hex	675	41.72 ± 0.69 bc	51.41 ± 1.60 cd	70.83 ± 1.38 ab	78.24 ± 1.94 b	82.45 ± 1.26 bc	87.56 ± 3.27 b
		1350	48.06 ± 3.04 b	58.12 ± 2.92 ab	69.06 ± 1.30 ab	82.20 ± 4.43 ab	90.02 ± 4.71 ab	92.71 ± 1.45 ab
	Clo	675	29.23 ± 4.60 d	43.49 ± 2.15 e	47.39 ± 5.61 d	52.00 ± 1.28 d	59.63 ± 2.46 e	64.80 ± 3.51 d
		1350	36.74 ± 4.36 cd	55.08 ± 1.35 bc	57.41 ± 4.56 cd	59.20 ± 2.39 d	68.36 ± 5.24d e	71.42 ± 2.12 cd

Note: Chive roots were drenched with 30% SC (clothianidin plus hexaflumuron, i.e., Clo + Hex) or 48% SC (clothianidin only, i.e., Clo) at 675 or 1350 g.a.i.ha^−1^ based on clothianidin. Different letters in each column indicates significant differences based on Tukey’s HSD test at *p* < 0.05 level.

## Data Availability

The data presented in this study are available in the article.
